# Tracing of *Listeria monocytogenes* Contamination Routes in Fermented Sausage Production Chain by Pulsed-Field Gel Electrophoresis Typing

**DOI:** 10.3390/foods7120198

**Published:** 2018-12-04

**Authors:** Valerij Pažin, Dean Jankuloski, Lidija Kozačinski, Vesna Dobranić, Bela Njari, Željka Cvrtila, José Manuel Lorenzo, Nevijo Zdolec

**Affiliations:** 1 Faculty of Veterinary Medicine, University of Zagreb, 10000 Zagreb, Croatia; valerij.pazin@vef.hr (V.P.); klidija@vef.hr (L.K.); vdobranic@vef.hr (V.D.); bnjari@vef.hr (B.N.); zcvrtila@vef.hr (Ž.C.); 2 Food Institute, Faculty of Veterinary Medicine, University “Ss. Cyril and Methodius” in Skopje, 1000 Skopje, Macedonia; djankuloski@fvm.ukim.edu.mk; 3 Centro Tecnológico de la Carne de Galicia, Rua Galicia Nº 4, Parque Tecnológico de Galicia, San Cibrao das Viñas, 32900 Ourense, Spain; jmlorenzo@ceteca.net

**Keywords:** physicochemical parameters, molecular detection assay, pulsed-field gel electrophoresis, foodborne pathogens

## Abstract

In this study, the presence of *Listeria monocytogenes* was assessed along the production process of fermented sausages in a small-scale facility. Following the isolation of the pathogen from the final product (ISO 11290-1), retrospective sampling was performed during the production of a new batch of sausages, including raw materials, casings, additives, sausage mixtures, sausages during fermentation, and environmental samples. *L. monocytogenes* was recovered from the following sampling points: the defrosting room and the cuttering, mixing, stuffing, and fermentation phases. Ten strains were isolated, molecularly confirmed as *L. monocytogenes* by means of a molecular detection system, and subjected to pulsed-field gel electrophoresis (PFGE) typing. On the basis of an unweighted pair group method with arithmetic mean (UPGMA) dendrogram from Ascl pulsotypes, the strains were indistinguishable (no band difference). The same pulsotypes of strains present in both batches of sausages, as well as in environmental samples, indicated the persistence of *L. monocytogenes* in the sausage production unit.

## 1. Introduction

*Listeria monocytogenes* is a foodborne pathogen that is particularly important when considering the safety of ready-to-eat food [1,2]. The persistence of *L. monocytogenes* strains in food production environments presents the most challenging issue in designing appropriate sanitation schemes [3]. The ability to produce biofilms is frequently reported in *L. monocytogenes* strains found in ready-to-eat meat-based food chains, including fermented sausage production environments [4]. Regarding the final products’ risk of containing pathogens, fermented sausages are generally known as microbiologically stabile and safe products [5]. However, the growth ability and reduction of *L. monocytogenes* depend on the type of fermented sausage and their physicochemical properties such as water activity, acidity, and starter cultures applied [6,7].

The possible sources of sausage contamination may include meat, casings, water, equipment, personnel, or working surfaces [8]. Following foodborne outbreaks or pathogens’ presence in final products, epidemiological studies or official controls are performed to detect the source and routes of contamination. In this respect, the characterization of *L. monocytogenes* strains and comparison of their genotypic fingerprints should be done by standardized and reliable methods. The pulsed-field gel electrophoresis (PFGE) method is considered a “gold standard” in comparing DNA fingerprint patterns of *L. monocytogenes* strains for epidemiological purposes, surveillance, and traceability through food production chains [9]. Some examples of PFGE typing during epidemiological studies of foodborne listeriosis were presented by Kleta et al. (2017) in Germany and Jensen et al. (2016) in Denmark [10,11]. 

The aim of this study was to assess the routes of contamination with *L. monocytogenes* in the fast-fermented sausage production of a small-scale plant located in Zagreb, Croatia. For this purpose, retrospective sampling was performed, and collected strains of *L. monocytogenes* were sub-typed by means of PFGE. The authors’ hypothesis was that the *L. monocytogenes* pulsotype recovered from the final product would be found in environmental or ingredient samples. 

## 2. Materials and Methods

The starting point of the present study was the detection of *L. monocytogenes* (ISO 11290-1) in a batch of fermented sausages produced in a small-scale meat processing facility. Sausages were produced from pork and beef, with added salt, dextrose, GDL E575, E450, E451, antioxidants E316, E315, E301, natural spices, spice extracts, and E250. The raw materials (pork, beef, and pork back fat) were defrosted, grounded, mixed with additives and spices, stuffed into natural swine casings, fermented and dried, and finally vacuum-packed and cold-stored. The shelf-life (90 days under vacuum, stored at 10–15° C) was estimated by the producer according to their self-control procedures, which included microbiological, chemical, and sensorial testing (data not provided).

### 2.1. Sampling and Analyses

Laboratory staff visited the facility to conduct an inspection of the production process and sampling of suspected points. Samples were taken from raw materials, casings, additives, and production surfaces during the production of sausages. The presence of *L. monocytogenes* was tested following ISO 11290-1, i.e., by using pre-enrichment and enrichment broth (Half-Fraser and Fraser, Oxoid, UK) and plating on ALOA agar (Biokar, Pantin, France). The newly produced batch of fermented sausages were tested for pH (pH 510 Eutech Instruments, Nijkerk, The Netherlands), salt (Mohr method), water activity (a_w_; HigroPalm AW1, Rotronic, Bassersdorf, Switzerland), and *L. monocytogenes* presence and count (ISO 11290-1 and ISO 11290-2). 

### 2.2. Molecular Confirmation of L. monocytogenes Strains

Selected strains (*n* = 10) of suspected *L. monocytogenes* were identified by API Listeria (bioMérieux, Marcy l’Etoile, France) and confirmed by means of *Listeria monocytogenes* molecular detection assay (MDA, 3M, St. Paul, MN, USA). For the MDA analysis, isolates were grown in Fraser broth (3M, St. Paul, MN, USA) for 24 h at 37 °C. After incubation, the protocol recommended by the producer was followed. The 3M MDA uses continuous isothermal amplification of nucleic acid sequences, while bioluminescence is used to detect the amplification. Presumptive positive results are reported in real time, while negative results are displayed after the assay is completed (75 min). In the assay, pyrophosphate ions, generated by the amplification of the targeted DNA, and a substrate are enzymatically converted into adenosine triphosphate (ATP) by ATP-sulfurylase. ATP reacts with luciferase to produce light, which is then detected by the 3M molecular detection instrument, indicating the presence of *L. monocytogenes* DNA (3M, St. Paul, MN, USA).

### 2.3. Pulsed-Field Gel Electrophoresis (PFGE) Typing of L. monocytogenes Strains

Genetic typing was performed by a standardized laboratory protocol for the molecular typing of *L. monocytogenes* with PFGE in one day (24 to 48 h) [12]. An analysis of the obtained patterns of restrictive endonuclease ApaI was made with FPQuest V.5.10. software. The comparison was based on the assessment of the band (line) for each form and a calculation using the Dice coefficient. The dendrogram type when testing the impressions was formed by pairs of non-measured groups using the unweighted pair group method with arithmetic mean (UPGMA). The tolerance of the position was set to 1.5%, while the optimization was 0.5%.

## 3. Results and Discussion

*L. monocytogenes* was recovered from several sampling points during the inspection of the fermented sausage production unit (Table 1). Insufficient cleaning of equipment and working surfaces was noted between the production of two types of sausages, i.e., dry sausages were produced immediately after semi-dry sausages in the same production unit using the same equipment. This mistake resulted in findings of *L. monocytogenes* in both semi-dry and dry sausage mixtures, as a result of similar raw materials used or cross-contamination via the equipment (cutter, stuffing machine).

**Figure 1:**
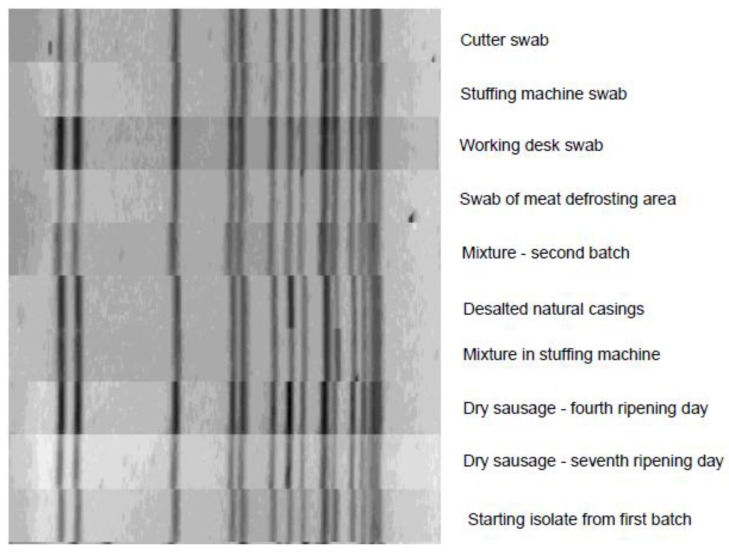
PFGE with ApaI enzyme images of the strains; dendrogram obtained using FPQUest (Biorad) software and Unweighted Pair Group Method with Arithmetic Mean (UPGMA), Dice (Opt: 0.50%) (Tol: 1.5–1.5%) (H > 0.0%; S > 0.0%) (0.0–100.0%)

Ten strains of *L. monocytogenes* were subjected to molecular confirmation by the MDA procedure, and all were confirmed as targeted species. In order to track the route of pathogen contamination, the strains were subjected to PFGE typing, the results of which are presented in Figure 1. On the basis of the UPGMA dendrogram from Ascl pulsotypes (Figure 1), our strains from this study (named in the dendogram) are indistinguishable (no band difference). It is interesting that the strain first isolated from the final product (from the first batch which preceded this retrospective study) shared the identical PFGE profile with newly isolated strains from the production unit and the corresponding sausage batch. The same PFGE profiles of *L. monocytogenes* were found in both semi-dry and fermented sausage mixtures, which indicates that the cross-contamination was probably due to the practice of processing the same raw material (swab of bag with frozen beef positive for pathogen) and to the contact of both mixtures in the production equipment (cutter and stuffing machine positive for pathogen).

The production of naturally contaminated sausages was continued, and the product was tested during fermentation (14 days) and storage (30 days). *L. monocytogenes* was present in high amounts until the seventh day of fermentation, together with higher water activity and usual pH values for this kind of product (Table 2). The pathogen was non-culturable at the end of fermentation (14th day) and during 30 days of storage. 

*L. monocytogenes* has been reported to be a contaminant of meat processing equipment and surfaces by other studies [13,14]. Ready-to-eat meat production facilities have been found to be sources of persistent *L. monocytogenes* strains due to the increased adhesion and biofilm capacity of the strains [15]. The contamination routes of foodborne pathogens may be difficult to trace; however, molecular typing has been proposed as a reliable tool for epidemiological purposes [16,17]. Recently, Véghová et al. [18] applied PFGE analysis in the molecular typing of *L. monocytogenes* strains from traditional meat production facilities and confirmed the reliability of the method to reveal persistent contamination over a number of years in the same facility. 

The persistence of *L. monocytogenes* was also reported in different small food businesses in Ireland, on the basis of similar PFGE patterns found during repeated sampling in the same facilities [19]. Leong et al. [20] confirmed contamination with persistent strains of *L. monocytogenes* in seven food processing facilities. Moreover, evidence of bacterial transfer from the processing environment to the food (the same PFGE pulsotype was found in both) was seen in four of the food processing facilities tested. Our small study, supported by PFGE strain typing, underlines the risk of *L. monocytogenes* cross-contamination in small-scale fermented sausage production and the need for strict preventive hygienic programs in hazard control. 

Following the obtained results, authors proposed changes to the producer’s standard operating procedures and revisions of their Sanitation Standard Operating Procedures (SSOPs), prerequisite programs, and HACCP system. Several points were highlighted and should be improved, including the procedure and control of raw material supply, the revision of SSOPs, the education of meat handlers regarding sanitation, and standard operating procedures in sausage production. It was also recommended that an *L. monocytogenes* monitoring plan be included in the Food Bussines Operator’s procedures of environmental samples control (swabs or abrasive sponges for equipment and surfaces). During the fermentation phase, the measurement of pH in sausages is essential to assess the course of fermentation and may be suggested as a critical control point.

## Figures and Tables

**Table 1:** Results of *Listeria monocytogenes* testing of raw materials, swabs, and sausage mixtures.

Samples	*L. monocytogenes* Presence
Fresh beef	Negative
Fresh pork	Negative
Frozen beef	Negative
Fat tissue	Negative
Swab of mixing machine	Negative
Salted natural swine casings	Negative
* Cutter swab	Positive
* Stuffing machine swab	Positive
* Working desk swab	Positive
* Swab of meat defrosting area	Positive
* Mixture—second batch	Positive
* Desalted natural casings	Positive
* Mixture in stuffing machine	Positive
* Dry sausage—fourth ripening day	Positive
* Dry sausage—seventh ripening day	Positive

*, the same labels of sampling points are listed in Figure 1, presenting the pulsed-field gel electrophoresis (PFGE) pulsotype of the representative isolate.

**Table 2:** Results of the physicochemical and microbiological analyses of naturally contaminated dry sausages during fermentation and storage.

Parameter	Days of Fermentation				Days of Storage	
	0	4	7	14	14	30
pH	5.37	5.12	5.15	5.23	5.33	5.43
a_w_	0.976	0.952	0.868	0.827	0.790	0.780
Salt (%)	2.5	2.56	3.05	3.75	3.42	3.30
*L. monocytogenes* (cfu/g)	600	200	<100	<100	<100	<100
*L. monocytogenes* (25 g)	Positive	Positive	Positive	Negative	Negative	Negative
